# Purity Determines the Effect of Extracellular Vesicles Derived from Mesenchymal Stromal Cells

**DOI:** 10.3390/cells9020422

**Published:** 2020-02-12

**Authors:** Maria Antònia Forteza-Genestra, Miquel Antich-Rosselló, Javier Calvo, Antoni Gayà, Marta Monjo, Joana Maria Ramis

**Affiliations:** 1 Cell Therapy and Tissue Engineering Group, Research Institute on Health Sciences (IUNICS), University of the Balearic Islands, Ctra Valldemossa km 7.5, 07122 Palma, Spain; maria.forteza@ssib.es (M.A.F.-G.); miquel.antich1@estudiant.uib.es (M.A.-R.); jcalvo@fbstib.org (J.C.); agaya@fbstib.org (A.G.); 2 Health Research Institute of the Balearic Islands (IdISBa), 07120 Palma, Spain; 3 Fundació Banc de Sang i Teixits de les Illes Balears (FBSTIB), 07004 Palma, Spain

**Keywords:** extracellular vesicles, ultracentrifugation, size exclusion chromatography, ATDC-5 cell line, gene expression, collagen, human umbilical cord mesenchymal stromal cells, FBS, conditioned media, purity

## Abstract

Extracellular vesicles (EVs) have been recently identified as vital components of cell-based therapies based on the observation that conditioned media from cultured stromal cells reproduce some of the beneficial effects of intact cells. In order to obtain clinically active EVs derived from Mesenchymal Stromal Cells (MSCs) different procedures have been reported in the literature. Usually, non-confluent cells are incubated with culture medium for 48 h either with EV-depleted Fetal Bovine Serum (FBS) or without FBS. Our aim was to compare the effects of EVs isolated by ultracentrifugation from human umbilical cord MSC conditioned media obtained using these two conditions: with EV-depleted FBS (UC) or without FBS (UC_w/o_) on the mRNA expression levels of extracellular matrix related genes using the mouse chondrogenic cell line ATDC-5. We observed a deleterious effect on chondrogenic cells treated with UC_w/o_, showing higher mRNA expression levels of different metalloproteinases and decorin (*Dcn*) and lower collagen (*Col1a1* and *Col2a1*) and aggrecan (*Acan*) mRNA levels. To elucidate whether this deleterious effect was induced by the EVs or by any proteins co-purified in the EV pellet, we used size exclusion chromatography (SEC) to further purify the EV pellet, obtaining an EV enriched fraction (EV or EV_w/o_) and a protein enriched fraction (Prot or Prot_w/o_). Our results pointed that the negative effect on the chondrogenic cell line was due to the contaminant proteins coisolated with the EVs by ultracentrifugation and not from the EVs themselves. Thus, these results highlight the importance of working with well purified EV preparations to specifically achieve their therapeutic effect.

## 1. Introduction

Mesenchymal Stromal Cells (MSCs) play an important role in cell therapy and in regenerative medicine due to their capability to differentiate in different cell types depending on the experimental or physiological conditions [1]. However, there are evidences that this beneficial effect cannot be attributed to cell survival and differentiation, and cells are rather thought to act in a paracrine manner [2]. This is based, on one hand, on in vivo studies showing low and transient stem cell engraftment and differentiation at injury sites [3,4,5,6,7] and, on the other hand, on the observation that some of the beneficial effects are reproduced when conditioned medium from cultured stem cells is used [8,9,10,11]. This paracrine effect exerted by stem cells depends not only on their capacity to secrete soluble factors [12], but also on the release of extracellular vesicles (EVs) [13,14,15].

EVs carry proteins, lipids and nucleic acids that have functional effects on other cells, thus, being in part responsible for the cell-to-cell communication of MSCs [16]. EV regenerative potential has been widely studied in different approaches including kidney, liver, heart, bone, cartilage or neural injuries, proving that EVs and their cargos are related to cell proliferation, cell regenerative mechanisms or prevention of apoptosis [17,18]. Moreover, EVs present the ability to cross biological barriers with the possibility to reach target cells easily, they are immunologically compatible and their membranes and cargo can be modified [19]. Hence, EVs are acquiring a potential role as drug carriers [17] and therapeutic agents [20]. In fact, currently different phase I and II clinical trials in different areas are being undertaken [21,22,23,24,25,26,27,28].

To obtain EVs from MSCs and use them in therapeutics, MSCs are harvested from culture media. However, there is no agreement on whether using cell culture media with or without Fetal Bovine Serum (FBS) to obtain EVs [29], since there exists a risk of contamination with EV derived from FBS when used as supplement in the cell culture media, even if this FBS has been previously EV-depleted as suggested by Shelke et al. [30]. The same risk exists when using human platelet lysate instead of FBS, since it also contains EVs [31]. Moreover, the market is developing media to be used for cell culture without the need of adding FBS although currently these solutions are not as widely used as conventional culture media.

Previous studies have shown that serum-free conditions lead to increased EV production with protein composition that differs from EVs isolated in serum-containing media [32] and in MISEV2018 it is recommended to avoid FBS if possible for EV production [33]. However, the therapeutic effects and safety of EVs secreted in these conditions have not been tested. In addition, cellular stress under FBS starvation conditions may increase contaminants, such as apoptotic bodies [34]. For these reasons, there are some divergences in protocols when EVs are isolated from culture media. It is not clear whether it is better to use or not EV-depleted FBS in cell culture media [35,36,37]. Additionally, there are a lot of different protocols to isolate EVs such as ultracentrifugation, which is the gold standard protocol, centrifugation with sucrose gradient, ultrafiltration, size exclusion chromatography (SEC) or precipitation with polymers [38,39]; obtaining different EV purity grade according to the used method. In fact, SEC allows to obtain enriched EVs isolates with a better purity grade compared to other approaches [40,41,42].

Thus our aim was to compare the effects of EVs isolated by ultracentrifugation from MSC conditioned media obtained using EV-depleted FBS or incubating the cells in the absence of FBS. Then, in view of our results, we explored whether purer EVs obtained by SEC may influence the EV effect.

## 2. Materials and Methods

### 2.1. Mesenchymal Stromal Cells Culture, EVs Production and Ultracentrifugation

Human Umbilical Cord Mesenchymal Stromal Cells (MSCs) were obtained from the IdISBa Biobanc, with the approval of the Ethical Committee of Balearic Islands (CEI-IB). MSCs were cultured in proliferative medium DMEM–low glucose (Biowest, Nuaillé, France) supplemented with 20% FBS Embryonic Stem Cells tested (Biowest) and 1% penicillin-streptomycin (Biowest). For EV production, MSCs were used between passages 6 and 14.

MSCs were grown in 75 cm^2^ flasks with proliferative medium until 60% of confluence. Then, cells were washed twice with PBS and proliferative medium was replaced by DMEM–low glucose medium supplemented with 1% penicillin-streptomycin and 20% EV-depleted FBS Embryonic Stem Cells tested to obtain conditioned medium or supplemented just with 1% penicillin-streptomycin to obtain conditioned medium without FBS. EV-depleted FBS was obtained by centrifugation at 120,000 × *g* (sw 32 Ti rotor, 38.5 mL polypropylene tubes, Beckman Coulter, Brea, CA, USA) for 18 h at 4 °C and supernatant filtered on 0.22 µm porous membrane (Sartorius, Goettingen, Germany) as previously described [30]. After 48 h of incubation with the cells, conditioned media with EV-depleted FBS and conditioned media without FBS were collected and centrifuged at 1500 × *g* for 15 min at 4 °C to remove cell debris. Then, supernatants were filtered through a 0.22 µm porous membrane and centrifuged at 10,000× *g* for 30 min at 4 °C to remove largest EVs. Supernatants were centrifuged at 120,000 × *g* for 18 h at 4 °C (as previously reported [43]) and pellets were suspended in 300 µL of PBS (Biowest) obtaining EVs from conditioned media with EV-depleted FBS (UC) and EVs from conditioned media without FBS (UC_w/o_). Three independent isolation experiments (using 12 different 75 cm^2^ flasks in each) were performed for EV isolation and then, the obtained EVs were pooled and aliquoted in order to avoid thawing-freezing cycles and stored at −80 °C until use. Pooling was needed to get enough amount in order to perform further studies as it is done with RNA samples for microarray experiments [44]. Pooling of samples can be used assuming that the measurements taken on the pool are equal to the average of the measurements taken on the individuals [45].

After collecting conditioned media, MSCs cultured with EV-depleted FBS and without FBS were checked: bright-side field images were taken and cell monolayer was used to evaluate cell surface profile (CD105-phycoeritrin (PE), CD90-fluorescein (FITC), CD73-PE, CD34-PE, CD45-FITC and HLA-DR-FITC, Thermo Fisher) [46] by standard flow cytometry as previously described [47].

### 2.2. Size Exclusion Chromatography

SEC of UC and UC_w/o_ samples was performed as previously described by Böing et al. [48]. Briefly, 14 mL of Sepharose CL-2B (Sigma-Aldrich, St. Louis, MO, USA) was stacked into 10 mL syringe—1.5 cm inner diameter × 7.5 cm column length (BD biosciences, Franklin Lakes, NJ, USA)—stuffed with nylon stocking (Calzedonia, Italy) and washed with 10 mL PBS.

One milliliter of UC or UC_w/o_ sample was loaded on SEC columns and eluted with PBS. Twenty fractions of 0.5 mL eluate were collected. For each fraction, total protein content was quantified and EV markers were identified by western blot. Fractions 6-12 enriched in EVs (EV and EV_w/o_) and 13–22 enriched in proteins (Prot and Prot_w/o_) were pooled and characterized.

### 2.3. Total Protein Quantification

Total protein content was quantified by BCA Protein Assay kit (Thermo Fisher, Waltham, MA, USA), using a bovine serum albumin standard included in the assay or by reading absorbance at λ = 280 nm with the NanoDrop spectrophotometer (NanoDrop Technologies, Wilmington, DE, USA).

### 2.4. Atomic Force Microscopy (AFM)

EV samples (1 µg protein) were diluted in a final volume of 200 µL PBS and 50 µL were adsorbed to freshly cleaved Ruby Muscovite mica (NanoAndMore GmbH, Germany) discs for 10 min. The discs were rinsed with deionized water and dried gently under nitrogen stream. Atomic force microscope (Veeco, Oyster Bay, NY, USA) in tapping mode and aluminum coated silicon probe tips (Mikromasch, Lady’s Island, SC, USA) were used for imaging. Topographic height, amplitude and phase images were recorded at 512 pixels × 512 pixels at a scanning rate of 1 Hz. Images were processed using NanoScope Image software (Veeco Metrology, NY, USA).

### 2.5. Transmission Electron Microscopy (TEM)

EV samples (10 µg protein) were mixed 1:1 with 4% formaldehyde (Sigma-Aldrich) and 10 µL were fixed on copper Formvar-Carbon coated grids (Ted Pella, CA, USA) for 20 min. These grids were washed with PBS and incubated with 1% glutaraldehyde (Sigma-Aldrich) for 5 min. Finally, grids were washed with deionized water.

To contrast samples, grids were stained with 2% phosphotungstic acid for 1 min and then air dried. Images of EVs were taken with transmission electron microscope Hitachi H600 (Hitachi, Tokyo, Japan) at 50 kV.

### 2.6. Nanoparticle Tracking Analysis (NTA) and Purity Ratio

The number of particles in EV samples was analyzed using a ZetaView^®^ Nanoparticle Tracking Analyzer device (Particle Metrix GmbH, Meerbush, Germany). Before each session, size and concentration of standard polystyrene beads (100 nm) were measured. Samples were diluted 1:2000 before analyzing 22 position tracks per video.

Once the number of particles in EV samples was set, purity ratio was calculated as described by Webber et al. [49] using the following Equation (1):

(1) PurityParticlesµg=ParticlemLµgmL

### 2.7. Western Blot

EV samples were prepared with non-reducing loading buffer (without β-mercaptoethanol) to detect tetraspanins presence and loaded with the same amount of protein (10 µg) in a 12% SDS-PAGE gels. Proteins were transferred onto nitrocellulose membrane (GE Healthcare, Pittsburgh, PA, USA) by humid transference, blocked with 10% dry skimmed milk (Central Lechera Asturiana, Asturias, Spain) in TBS containing 10% Tween-20 (Scharlab, Barcelona, Spain) and incubated overnight at 4 °C with the following primary antibodies: anti-human CD9 monoclonal antibody (clone Ts9 diluted 1:2000, Thermo Fisher) and anti-human CD63 monoclonal antibody (clone TS63, diluted 1:2000, Abcam, Cambridge, UK). Then, membranes were incubated for 1 h with HRP-coupled secondary antibody (Thermo Fisher) diluted 1:2000. For membrane exposure, membranes were incubated with Clarity Western ECL Substrate (Bio-Rad, Hercules, CA, USA) and chemiluminescence was detected with C-DiGit^®^ Blot scanner (LI-COR Biosciences, Lincoln, NE, USA). Images were processed with Image Studio Digits Software version 4.0 (LI-COR Biosciences).

In order to confirm the correct transferring of the proteins, before blocking, membranes were then incubated with 0.2% (*w*/*v*) Ponceau S (Sigma-Aldrich) in 3% (*v*/*v*) acetic acid solution (Sigma-Aldrich) for 5 min. Then, membranes were washed with deionized water. After taking images, membranes were finally washed with TBS for 5 min.

### 2.8. Chondrogenic Cell Line Culture: Functional Study

ATDC-5 cells (25,000 cells/well) were seeded in 48 well plates with DMEM-F12 medium (Biowest) supplemented with 5% FBS Premium (Biowest), 1% penicillin-streptomycin, supplemented with 10 µg/mL transferrin (Sigma-Aldrich) and 5 ng/mL sodium selenite (Sigma-Aldrich). When cells reached confluence 10 µg/mL insulin (Sigma-Aldrich) were added to the medium to induce cell differentiation. Medium was refreshed every other day.

At day 15, cells were incubated with DMEM-F12 medium supplemented with 5% EV-depleted FBS Premium, 1% penicillin-streptomycin, 10 µg/mL transferrin and 5 ng/mL sodium selenite, and treated for 48 h either with one dose of UC (5 µg protein/1.51 × 10^7^ particles), UC_w/o_ (5 µg protein /1.37 × 10^9^ particles), EV (1.37 × 10^9^ particles), EV_w/o_ (1.37 × 10^9^ particles), Prot (5 µg protein) and Prot_w/o_ (5 µg protein). Two independent experiments were performed using 6 replicates in each study.

### 2.9. Lactate Dehydrogenase Activity

Lactate dehydrogenase (LDH) activity was measured from cell culture media after 48 h of treatment, following the manufacturer’s instructions (Cytotoxicity Detection kit, Roche Diagnostics, Manheim, Germany). Data were presented relative to cells treated with vehicle control (low control, 0% of cell death) and cells treated with 1% Triton X-100 (high control, 100% cell death). Cytotoxicity percentage was calculated using the following Equation (2):

(2) $$\mathrm{Citotoxicity}\left(\%\right)=\frac{\left(\mathrm{exp. value}-\mathrm{low}\;\mathrm{control}\right)}{\left(\mathrm{high}\;\mathrm{control}-\mathrm{low}\;\mathrm{control}\right)}\cdot 100$$

### 2.10. Cell Metabolic Activity

Metabolic activity was measured using PrestoBlue^TM^ Cell Viability Reagent (Life Technologies, Carlsbad, CA) after 48 h of treatment following the manufacturer’s instructions. Absorbance data were normalized to the control group that was set as 100% of metabolic activity with the following Equation (3):

(3) $$\mathrm{Metabolic}\;\mathrm{activity}\left(\%\right)=\frac{\left(\mathrm{ODU}570\mathrm{nm}-\mathrm{ODU}600\mathrm{nm}-\mathrm{ODUblank}\right)}{\mathrm{mean}\;\mathrm{ODUControl}}\cdot 100$$

### 2.11. Collagen Quantification

Collagen quantification by Sirius red staining was performed as previously described by Gómez-Florit et al. [50]. Briefly, after 48 h of treatment, ATDC-5 cells were washed with PBS and dried overnight at 37 °C in a humidified atmosphere. A solution of 0.1% Sirius red F3BA (Sigma-Aldrich) in saturated picric acid (Sigma-Aldrich) was used to stain collagen for 1 h. A wash with 10 mM HCl (Scharlab, Barcelona, Spain) was performed to remove unbound dye. Then, dye was solubilized with 100 mM NaOH (Scharlab) and absorbance was measured at 540 nm. Results were compared with a calfskin collagen standard curve included in the assay.

### 2.12. RNA Isolation and Real-Time RT-PCR Analysis

Total RNA isolation was performed using Tripure^®^ (Roche Diagnostics) and quantified at λ = 260 nm by NanoDrop spectrophotometer. To obtain cDNA, the same amount of RNA (500 ng) was reverse transcribed at 42 °C for 60 min using High Capacity RNA-to-cDNA kit (Applied Biosystems, Foster City, CA, USA).

Real-time PCR was performed for several target genes and two reference genes (Table 1) with the Lightcycler480^®^ (Roche Diagnostics) using SYBR green detection. For cDNA amplification, a pre-incubation cycle of 5 min at 95 °C was followed by 45 cycles of cDNA denaturation for 10 s at 95 °C, an annealing step of 10 s at 60 °C and finally an extension step for 10 s at 72 °C. After each cycle, fluorescence was measured at 72 °C. Water (Sigma-Aldrich) was used as negative control without cDNA. To allow relative quantification after PCR, standard curves were constructed from standard reactions for each target and reference genes. The crossing point readings for each sample were used to calculate the amount of either the target or the reference relative to a standard curve, using the Second Derivative Maximum Method provided by the LightCycler480^®^ analysis software version 1.5 (Roche Diagnostics). All samples were normalized by the mean of the expression levels of reference genes and changes were related to the control groups that were set to 100%.

### 2.13. Statistical Analysis

Two independent experiments were performed, and values represent either mean values with minima and maximum or mean ± standard deviation (SD), *n* = 11 or *n* = 12. The Kolmogorov–Smirnov test was used to assume parametric or non-parametric distribution for the normality tests. Differences between groups with parametric distribution were assessed by ANOVA followed by Bonferroni test as a post-hoc. When data were non-parametric, the Kruskal–Wallis test was assessed. Results were considered statistically significant at *p* < 0.05. SPSS program for Windows, version 25.0 (SPSS Inc., Chicago, IL, USA) was used.

## 3. Results

### 3.1. Characterization of MSC-Derived EVs Isolated by Ultracentrifugation

Conditioned media from MSCs cultured with EV-depleted FBS and without FBS was used to isolate EVs. On the one hand, MSCs analyzed by flow cytometry did not express CD34, CD45 and HLA-DR, whereas they were positive for CD105, CD90 and CD73, showing a typical profile of MSCs [46] (Supplementary Materials; Figures S1–S3) and morphology was not different as shown in bright-side field images (Supplementary Materials; Figure S4).

**Figure 1:**
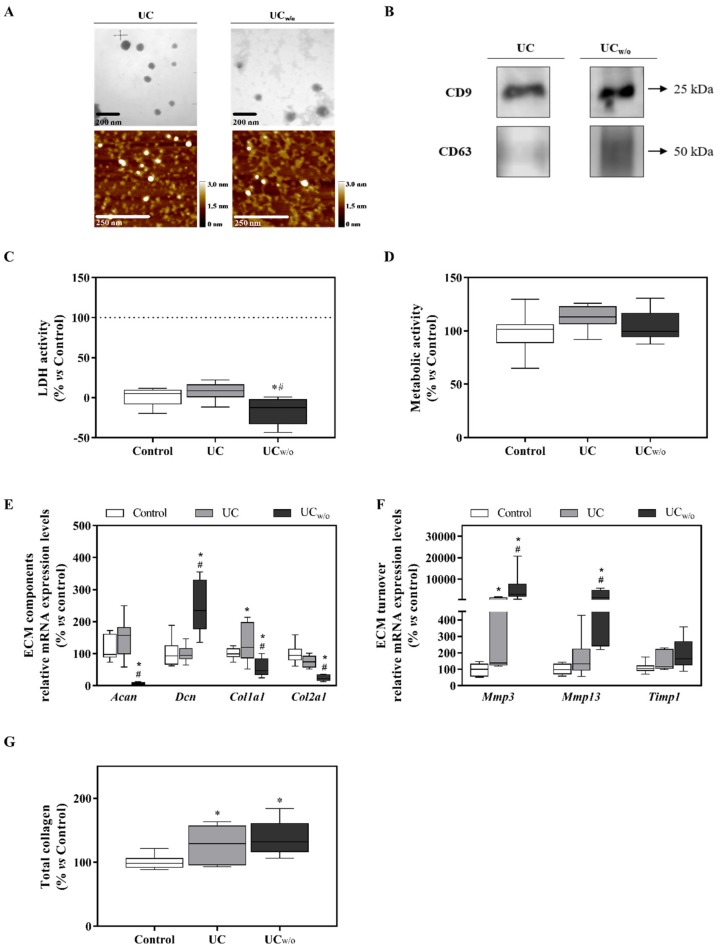
UC and UC_w/o_ isolates characterization and their effects on the ATDC-5 chondrogenic cell line. (**A**) Representative TEM and AFM images for each group of EVs with their respective scale bars. (**B**) Typical EV markers (CD9 and CD63) identified by western blot, loading the same amount of protein for both samples. (**C**) Lactate dehydrogenase activity was measured from cell culture media of ATDC-5 after 48 h of treatment with UC or UC_w/o_. Negative control (0%) was culture media from cells treated with the vehicle control. Positive control (100%) was culture media from cells treated with 1% Triton X-100. (**D**) Metabolic activity measured after 48 h of treatment. Data were normalized to the vehicle control group that was set to 100%. (**E**,**F**) mRNA expression levels of ECM components (*Acan*, *Dcn*, *Col1a1* and *Col2a1*) genes or ECM turnover genes (*Mmp3*, *Mmp13* and *Timp1*) of ATDC-5 after treatment with UC or UC_w/o_ for 48 h. Data represent fold changes of target genes normalized to reference genes (*Rn18s* and *Gapdh*) and relative to group control that was set as 100%. (**G**) Total collagen deposition in ATDC-5 cells treated with UC or UC_w/o_ for 48 h. Data were normalized to the vehicle control group that was set to 100%. Values presented in box and whisker plots represent the median, minima and maxima of two independent experiments (*n* = 11). Results were statistically compared by ANOVA and Bonferroni as post hoc for metabolic activity and *Col2a1* mRNA expression levels; and by Kruskal–Wallis for *Acan*, *Dcn*, *Col1a1*, *Mmp3* and *Mmp13* mRNA expression levels and total collagen deposition: * *p* < 0.05 versus the control; ^#^ *p* < 0.05 versus UC.

On the other hand, EV pellets (UC and UC_w/o_) were isolated from conditioned medium with EV-depleted FBS and conditioned medium without FBS by ultracentrifugation, which is considered a gold standard protocol [38]. EVs presence was confirmed by TEM and AFM as shown in Figure 1A. As shown in Table 2, EVs from UC_w/o_ were larger than EVs from UC, while higher protein content was quantified for UC compared to UC_w/o_ isolates, having this one a higher purity ratio as calculated following Webber et al. [49] parameters. For both EVs isolates, typical EV markers (CD9 and CD63) were identified by western blot (Figure 1B). Furthermore, when evaluating the negative controls, neither cytochrome C (a mitochondria marker) nor bovine serum albumin (as marker of FBS contaminant) was detected in these isolates, showing no presence of contamination due to the isolating process (Supplementary Materials; Figure S5).

### 3.2. Functional Study: UC and UC_w/o_

To evaluate the effect exerted by EVs isolated by ultracentrifugation from different conditions on cells, a functional study was performed using the ATDC-5 chondrogenic cell line. Cells were differentiated for 15 days and then treated for 48 h with the isolated EVs. As shown in Figure 1D, no differences were found as regards to metabolic activity, however, UC_w/o_ induced lower LDH activity released to the cell culture medium compared to UC or control (Figure 1C).

Different genes related with extracellular matrix (ECM) composition and turnover were studied to evaluate the effect of treatments in their mRNA expression levels (Figure 1E,F). On one hand, UC_w/o_ treatment had a deleterious effect on *Acan*, *Col1a1* and *Col2a1* expression levels, which decreased significantly compared to control and treatment with UC. However, *Dcn* expression levels increased compared to control and UC treatment. On the other hand, UC_w/o_ treatment significantly increased mRNA expression levels for *Mmp3* and *Mmp13* compared to control and UC treatment, while no differences were shown for *Timp1*.

Moreover, higher collagen deposition was found for both treated groups compared to control (Figure 1G).

### 3.3. EV and Protein Fractions Characterization

Since a deleterious effect was observed in the UC_w/o_ treated group, and in order to elucidate whether this effect was induced by the EVs themselves or by any proteins co-purified with the EV pellet, we used SEC in order to obtain an EV enriched fraction (EV or EV_w/o_) pooled from fractions 6-12 and a protein enriched fraction (Prot or Prot_w/o_) pooled from fractions 13-22 (individual characterization of each fraction is shown in supplementary data; Figures S6 and S7).

**Figure 2:**
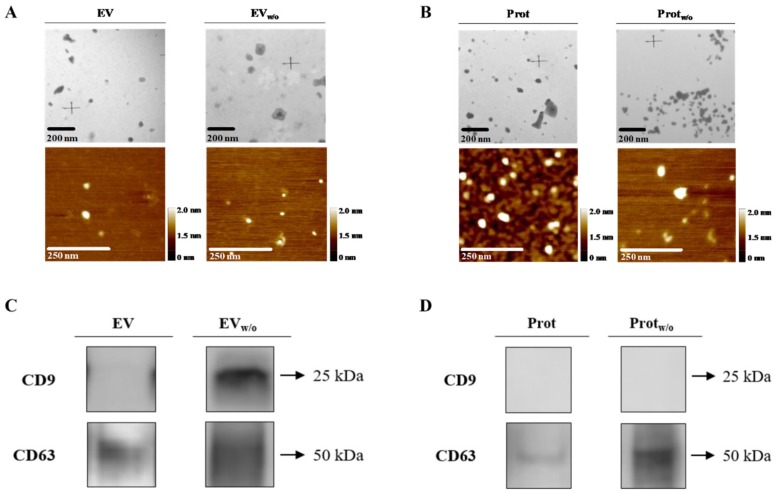
EV, EV_w/o_, Prot and Prot_w/o_ isolates characterization by TEM, AFM and western blot. (**A**,**B**) Representative TEM and AFM images for each group of EVs or pooled protein fractions with their respective scale bars. (**C**,**D**) Typical EV markers (CD9 and CD63) identified by western blot from EV, EV_w/o_ samples or Prot and Prot_w/o_, loading the same amount of protein for both group samples.

As expected, both EV fractions obtained after SEC presented a high purity ratio, even EV isolates from UC (EV), which were classified as unpure (Table 2). Following this purification step, a higher number of EVs was obtained in EV_w/o_, showing higher size compared to EV. Furthermore, typical EV marker tetraspanins appeared: CD9 in EV_w/o_ and CD63 in both EV enriched fractions (Figure 2C) in contrast to protein enriched fractions (Figure 2D). As found on UC samples, neither cytochrome C nor bovine serum albumin was detected for any of the evaluated fractions (Figure S5).

Prot and Prot_w/o_ were also characterized to confirm that, although, some EVs were also present as shown by TEM and AFM (Figure 2B), a lower number of particles/mL were quantified (Table 2). Protein content of Prot_w/o_ was statistically lower than Prot and purity ratio was in the high purity range for Prot_w/o_ and low purity range for Prot. Tetraspanin CD63 was identified, too, but showing a very low signal in Prot compared to Prot_w/o_ (Figure 2D).

### 3.4. Functional Study with the SEC Purified Fractions

**Figure 3:**
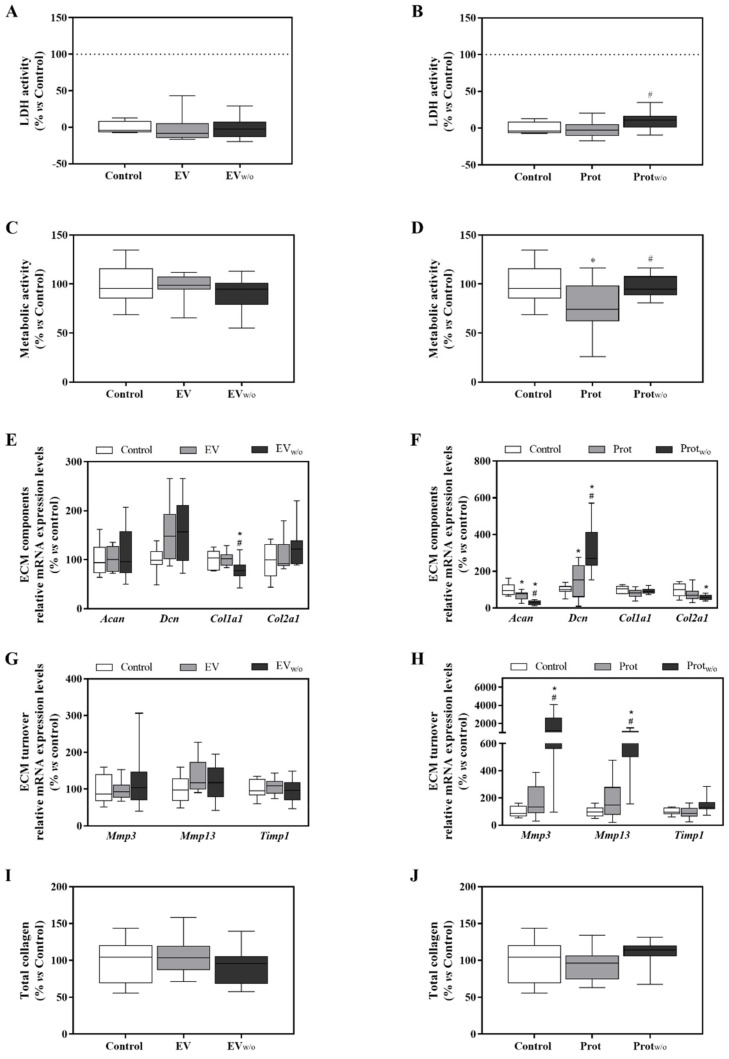
EV, EV_w/o,_ Prot and Prot_w/o_ isolates effects on ATDC-5 chondrogenic cell line. (**A**,**B**) Lactate dehydrogenase activity was measured from cell culture media of ATDC-5 after 48 h of treatment with EV and EV_w/o_ or Prot and Prot_w/o_. Negative control (0%) was the culture media from cells treated with the vehicle control. Positive control (100%) was the culture media from cells treated with 1% Triton X-100. (**C**,**D**) Metabolic activity measured after 48 h of treatment. Data were normalized to the vehicle control group that was set to 100%. (**E**–**H**) mRNA expression levels of ECM components genes (*Acan*, *Dcn*, *Col1a1* and *Col2a1*) or ECM turnover genes (*Mmp3*, *Mmp13* and *Timp1*) of ATDC-5 after treatment with EV and EV_w/o_ or Prot and Prot_w/o_ for 48 h. Data represent fold changes of target genes, normalized to reference genes (*Rn18s* and *Gapdh*) and relative to the group control that was set as 100%. Values presented in box and whisker plots represent the median, minima and maxima of two independent experiments (*n* = 11) Results in subfigures **A**,**C**–**E**,**G** and **I** were statistically compared by ANOVA and Bonferroni as post hoc for cytotoxicity, metabolic activity, *Acan* mRNA expression levels and total collagen deposition; and by Kruskal–Wallis for *Dcn*, *Col2a1*, *Mmp3* and *Mmp13* mRNA expression levels: * *p* < 0.05 versus the control; ^#^ *p* < 0.05 versus Prot. Results in subfigures **B**,**D**,**F**,**H** and **J** were statistically compared by ANOVA and Bonferroni as post hoc for *Acan*, *Col1a1*, *Mmp3*, *Mmp13* and *Timp1* mRNA expression levels; and by Kruskal–Wallis for *Col2a1* mRNA expression levels: * *p* < 0.05 versus Control; ^#^ *p* < 0.05 versus EV.

Once purer EVs were obtained, a functional study was performed to clarify if the deleterious effect found for UC_w/o_ was due to the EVs secreted by the cells when cultured without FBS or if such an effect was caused by proteins co-purified with the EVs. As shown in Figure 3A,C,I, no significant changes in LDH activity, metabolic activity or total collagen deposition were observed when chondrogenic cells were treated with EV or EV_w/o_; and only a slight decrease in *Col1a1* mRNA levels was found in cells treated with EV_w/o_ (Figure 3E). On the contrary, a similar response to UC_w/o_ was found for cells treated with Prot_w/o_; inducing a down-regulation of the ECM components *Acan* and *Col2a1*, and an up-regulation of *Dcn*, *Mmp3* and *Mmp13* and *Timp1* mRNA levels (Figure 3F,H).

## 4. Discussion

Over the last years, the interest on MSC-derived EVs has increased. EV isolation from MSCs requires a protocol that assures that these EVs can have a therapeutic use. Hence, it is important to decide whether to use or not EV-depleted FBS when isolating MSC derived EVs. Nevertheless, our results demonstrate that the purity of EV preparations is more important than the usage or not of EV-depleted FBS in determining their final effect in chondrocytes.

When isolating EVs from cell culture conditioned media, different procedures have been reported in the literature. Usually, non-confluent cells are incubated with culture medium for 48 h either with EV-depleted FBS or without FBS, though, as far as we are concerned, a direct comparison of the effect of EVs obtained by these two protocols has not been previously reported. On one hand, the use of FBS, even when having been previously EV-depleted, entails a risk of contamination with FBS-derived EVs; or when using FBS alternatives such as human platelet lysate, since it also contains EVs [30,31,45]. On the other hand, even if MISEV2018 [33] recommends serum-free cell culture to obtain EVs, using serum-free conditions increases the risk of contaminants due to cellular stress [34]. In fact, here, we show evidences that cells cultured without FBS supplementation need to be highly purified to avoid the effect of co-purified proteins that, in some cases, might be deleterious.

We observed a higher amount of protein content in UC, yielding a sample with a poor purity ratio. This finding was not surprising, since the proteins coming from the FBS supplement are expected to be in the medium. Interestingly, although the EVs of the UC_w/o_ group showed higher purity and lower protein contamination, this group showed a deleterious effect on the chondrocyte cell line, down-regulating mRNA expression levels of ECM related-components and up-regulating mRNA expression levels of different matrix-metalloproteinases, thus this effect would lead to collagen degradation, and thus could contribute to tissue break-down. It has previously been reported that protein presence and composition in the neuroblastoma cells-derived EVs may be different when obtained from cells cultured in medium with EV-depleted FBS or medium without FBS [32]. Furthermore, contamination with proteins generally occurs when cells are cultured in serum-containing media but also in serum-free conditions, mainly because cells produce soluble proteins that may interfere in later analysis [51].

Some studies have shown that EVs harvested under stress conditions have a stress effect on non-stressed cells due to their cargo [52,53,54]. Thus, our first thought was that the UC_w/o_ group had EVs with a cargo that was inducing the deleterious effect on chondrocytes. However, using ultracentrifugation as EV isolation method we could not discard the fact that stress inducing contaminants were co-precipitated with our EV sample. In fact, some studies showed that centrifugations at 100,000 × *g* form protein aggregates that co-precipitate with the EVs, leading to low purity samples, or even when harvesting the UC pellet some remaining supernatant, extremely rich in proteins, could be taken affecting the purity of the EV preparation. The yield obtained using ultracentrifugation is also low, and EVs can be damaged after centrifugation at that speed [44,55,56]. Even if ultracentrifugation has been considered for years the gold standard protocol, currently, extensive research on EVs has shown that other protocols for EV isolation allow us to obtain purer EVs and assure their usage in therapeutics. In view of our first results, we next performed SEC as a strategy to obtain purer EVs, as it was done in previous studies [44]. This technique allows the separation of EV fractions from protein fractions, thus obtaining purer and more functional EVs without altering their characteristics [41,47]. In fact, EV and EV_w/o_ fractions showed lower levels of protein content compared to UC or UC_w/o_, yielding as well to purer EV samples. In addition, what is more important, the same effect on chondrogenic cells was observed for EV and EV_w/o_ treatments, and, at the same time, the deleterious effect pattern caused by UC_w/o_ appeared in cells treated with Prot_w/o_. Thus, pointing that the deleterious effect is not caused by EV_w/o_ cargo but instead by contamination with co-precipitated elements when they are isolated by ultracentrifugation, this contamination can be removed by using a purification method such as SEC. These results were unexpected taking into account previous reports isolating EVs from conditioned media using EV-depleted FBS by ultracentrifugation, which showed that the deleterious effects were caused by EV cargo and not by the presence of stressing proteins on the EV fraction [52,53,54].

## 5. Conclusions

Our results show that the negative effect on the chondrogenic cell line is due to the contaminant proteins co-isolated with the EVs by ultracentrifugation and not from the EVs themselves, highlighting the importance of working with well purified EV preparations to specifically achieve their therapeutic effect.

From the results obtained in this study we show evidences that cells cultured without FBS supplementation need to be highly purified to avoid the effect of co-purified proteins that, in some cases, might be deleterious.

## Figures and Tables

**Table 1:** Primers of reference and target genes used in real-time PCR.

Related Function	Gene	Primer Sequence (5′→3′)	Product Size (bp)	GeneBank Accession Number
ECM component	Collagen type I, alpha 1 (*Col1a1*)	S: AGAGCATGACCGATGGATTC A: CCTTCTTGAGGTTGCCAGTC	177	NM_007742.4
ECM component	Collagen type II, alpha 1 (*Col2a1*)	S: CCTGCAGGTGCTTCTGGTAA A: TAAAGCCAGCAATGCCAGGT	184	NM_031163.3
ECM component	Decorin (*Dcn*)	S: TTGATGCACCCAGCCTGAAA A: TGTGAAGGTAGACGACCTGG	195	NM_001190451.2
ECM component	Aggrecan (*Acan*)	S: TGACGGACACTCTCTGCAAT A: CACGGTGCCCTTTTTACACG	163	NM_007424.2
ECM turnover	Matrix metalloproteinase-3 (*Mmp3*)	S: TAAAGACAGGCACTTTTGGCG A: GGAGACCCAGGGTGTGAATG	218	NM_010809.2
ECM turnover	Matrix metalloproteinase-13 (*Mmp13*)	S: GCCATTACCAGTCTCCGAGG A: GAGCCCAGAATTTTCTCCCTCT	196	NM_008607.2
ECM turnover	Metallopeptidase inhibitor 1 (*Timp1*)	S: GATCGGGGCTCCTAGAGACA A: AGCCCTTATGACCAGGTCCG	168	NM_011593.2
Reference gene	18s ribosomal RNA (*Rn18s*)	S: GTAACCCGTTGAACCCCATT A: CCATCCAATCGGTAGTAGCG	151	NR_003278.3
Reference gene	Glyceraldehyde 3-phosphate dehydrogenase (*Gapdh*)	S: ACCCAGAAGACTGTGGATGG A: CACATTGGGGGTAGGAACAC	171	NM_008084.3

**Table 2:** Particles characterization in terms of quantity, size and purity.

Sample	Number Particles (Particles/mL)	NTA Size	Protein Content (µg/µL)	Purity Ratio (Particles/µg) ^§^
UC	6.30 × 10^10^	150 ± 61.2	21.4	2.94 × 10^9^
UC_w/o_	2.20 × 10^11^	154 ± 59.8 *	0.800 *	2.75 × 10^11^
EV	1.20 × 10^11^	122 ± 73.5	0.050	2.40 × 10^12^
EV_w/o_	6.40 × 10^10^	147 ± 78.4 *	0.080	8.00 × 10^11^
Prot	7.50 × 10^9^	192 ± 107.1	3.52	2.13 × 10^9^
Prot_w/o_	5.10 × 10^10^	187 ± 119.7	0.270^*^	1.89 × 10^11^

§As described by Webber et al. [49]: High Purity: >3 × 10^10^ particles/µg; low purity: 2 × 10^9^–2 × 10^10^ particles/µg; unpure: <1.5 × 10^9^. Results were statistically compared by Student *t*-test: * *p* < 0.05 UC vs. UC_w/o_, EV vs. EV_w/o_ or Prot vs. Prot_w/o_.

## Supplementary Materials

The following are available online at https://www.mdpi.com/2073-4409/9/2/422/s1, Figure S1: MSC profile by cytometry after conditioning for 48 h in proliferative media with 20% FBS. Figure S2: MSC profile by cytometry after conditioning for 48 h in medium with 20% EV-depleted FBS. Figure S3: MSC profile by cytometry after conditioning for 48 h in medium without FBS. Figure S4: Representative bright-field images of MSCs in culture under all conditions. Figure S5: EV markers on UC, UC_w/o_, EV, EV_w/o_, Prot and Prot_w/o_ samples and cell lysates. Figure S6: Presence of proteins and EV marker in SEC fractions collected from UC and UC_w/o_ samples. Figure S7: Total protein quantification of SEC fractions collected from UC and UC_w/o_ samples.
