# Supplementary material for: Purity Determines the Effect of Extracellular Vesicles Derived from Mesenchymal Stromal Cells
Source: Cells. 2020 Feb 12;9(2):422. doi: 10.3390/cells9020422 (PMC7072280; doi:10.3390/cells9020422)
Supplement: Supplementary file 1 [file cells-09-00422-s001.pdf]

cells MDPI

Article

Purity determines the effect of Extracellular Vesicles derived from Mesenchymal Stromal Cells (Supplementary data)

Maria Antònia Forteza-Genestra [†,1,2], Miquel Antich-Rosselló [†,1,2], Javier Calvo [1,2,3], Antoni Gayà [1,2,3]; Marta Monjo [1,2,*], Joana Maria Ramis [1,2,*]

[1] Cell Therapy and Tissue Engineering Group, Research Institute on Health Sciences (IUNICS), University of the Balearic Islands, Ctra Valldemossa km 7.5, 07122 Palma, Spain; maria.forteza@ssib.es (M.A.F.-G.); miquel.antich1@estudiant.uib.es (M.A.-R.); jcalvo@fbstib.org (J.C.); agaya@fbstib.org (A.G.)
[2] Health Research Institute of the Balearic Islands (IdISBa), 07120, Palma, Spain
[3] Fundació Banc de Sang i Teixits de les Illes Balears (FBSTIB), 07004, Palma, Spain
* Correspondence: marta.monjo@uib.es (M.M.), joana.ramis@uib.es (J.M.R.); Tel.: +34 971 25 99 60 (M.M.), +34 971 25 96 07 (J.M.R.)
† Both authors contributed equally to this work

Received: 15 January 2020; Accepted: 10 February 2020; Published: date

1. Materials and methods

MSC bright-field imaging and phenotyping by flow cytometry

Bright-field images were taken with optical microscope Nikon Eclipse TS100 (Nikon, Tokyo, Japan) after 48 h before phenotyping by standard flow cytometry to determine MSC surface profile. Briefly, cells growth in different conditions (proliferative media, media with EV-depleted FBS and media without FBS) for 48 hours were resuspended and marked at concentration of $1·10^5$ cells/500 µL. For phenotypic analysis, cells were single labelled with fluorescein isothiocyanate (FITC) or phycoerythrin (PE) conjugated monoclonal antibodies: CD105-PE, CD90-FITC, CD73-PE, CD34-PE, HLA-DR-FITC (eBioscience, Thermo Fisher Scientific) and CD45-FITC(Life Technologies, Thermo Fisher Scientific). After labelling, cells were incubated for 10 minutes at 4 °C in darkness. Then, cells were washed with FC buffer (PBS with 20% human AB serum at pH 7.2) to remove antibodies not labelled to the cells. Finally, Guava® easyCyte™ System flow cytometer (Millipore, Burlington, MA, USA) was used to measure simultaneous forward (FSC) and side scatter (SSC), and to analyse the multiparametric fluorescent phenotypic marker signals. Ten thousand events were acquired for each analysis. Sample histogram elaboration was performed with Guava InCyte software to assess fluorescent distribution.

Western blot

EV samples and MSCs were lysed in ice-cold RIPA lysis buffer containing protease inhibitor (Roche Diagnostics) at the concentration recommended by the manufacturer. Non-lysate samples were prepared with non-reducing loading buffer (without β-mercaptoethanol) to detect tetraspanins presence. Cell lysate (cell lysate and cell lysate$_{w/o}$) and EV lysate samples were prepared with reducing loading buffer (with β-mercaptoethanol) to detect cytochrome C and bovine serum albumin. All samples were loaded with the same amount of protein (10 µg) in a 10 or 12% SDS-PAGE gels. Proteins were transferred onto nitrocellulose membrane by humid transference, blocked with 10% dry skimmed milk in TBS containing 10% Tween-20 and incubated overnight at 4 °C with the following primary antibodies: anti-human CD9 monoclonal antibody (clone Ts9 diluted 1:2,000), anti-human CD63 monoclonal antibody (clone TS63, diluted 1:2,000),

anti-cytochrome C monoclonal antibody (clone 37BA11, diluted 1:2,000, Abcam) and anti-Bovine Serum Albumin monoclonal antibody (clone 2A3E6, diluted 1:1,000, Santa Cruz, Dallas, TX, USA). Then, membranes were incubated for 1 h with HRP-coupled secondary antibody diluted 1:2,000. For membrane exposure, membranes were incubated with Clarity Western ECL Substrate and chemiluminescence were detected with C-DiGit® Blot scanner. Images were processed with Image Studio Digits Software version 4.0.

In order to confirm the correct transferring of the proteins, before blocking, membranes were then incubated with 0.2% (w/v) Ponceau S in 3% (v/v) acetic acid solution for 5 min. Then, membranes were washed with deionized water. After taking images, membranes were finally washed with TBS for 5 min.

2. Results

MSC characterization after conditioned media collection

Figure S1, S2, S3 and S4 show representative bright-field images of MSCs in culture after collecting conditioned media under all conditions and cell surface profile plots obtained by flow cytometry. Cells did not express CD34, CD45, and HLA-DR, while being positive for CD105, CD90, and CD73, showing a typical profile of MSCs [46]. Moreover, similar % of viability for all the groups was confirmed (94.8% for control, 95.3% for MSC conditioned without FBS and 95.8% for MSCs with FBS).

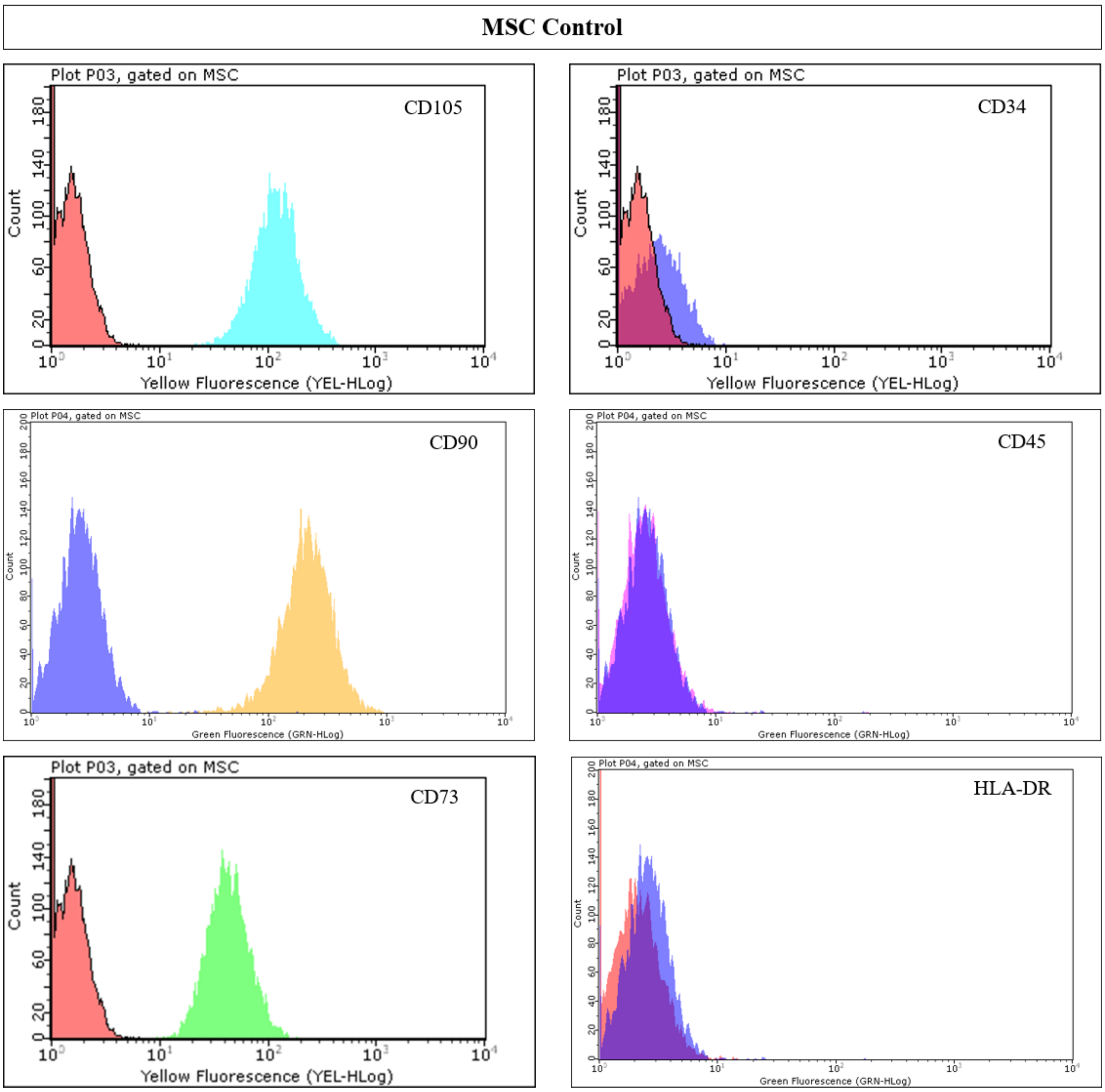

Figure S1: MSC profile by cytometry after conditioning for 48 hours in proliferative media with 20% FBS. A typical profile of MSCs is shown after labelling cells with conjugated monoclonal antibodies: CD105-PE, CD90-FITC, CD73-PE, CD34-PE, CD45-FITC and HLA-DR-FITC.

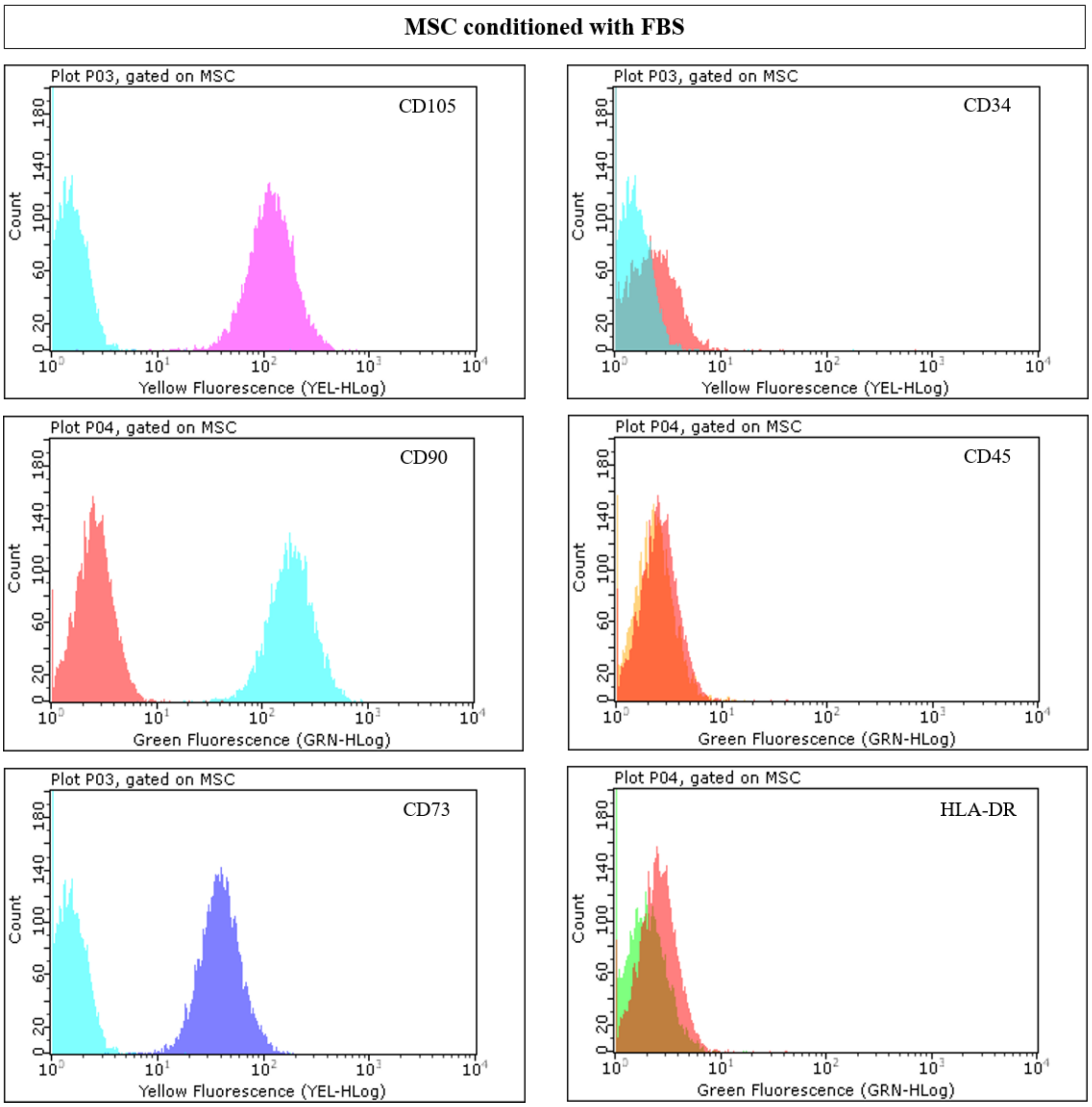

Figure S2: MSC profile by cytometry after conditioning for 48 hours in medium with 20% EV-depleted FBS. A typical profile of MSCs is shown after labelling cells with conjugated monoclonal antibodies: CD105-PE, CD90-FITC, CD73-PE, CD34-PE, CD45-FITC and HLA-DR-FITC.

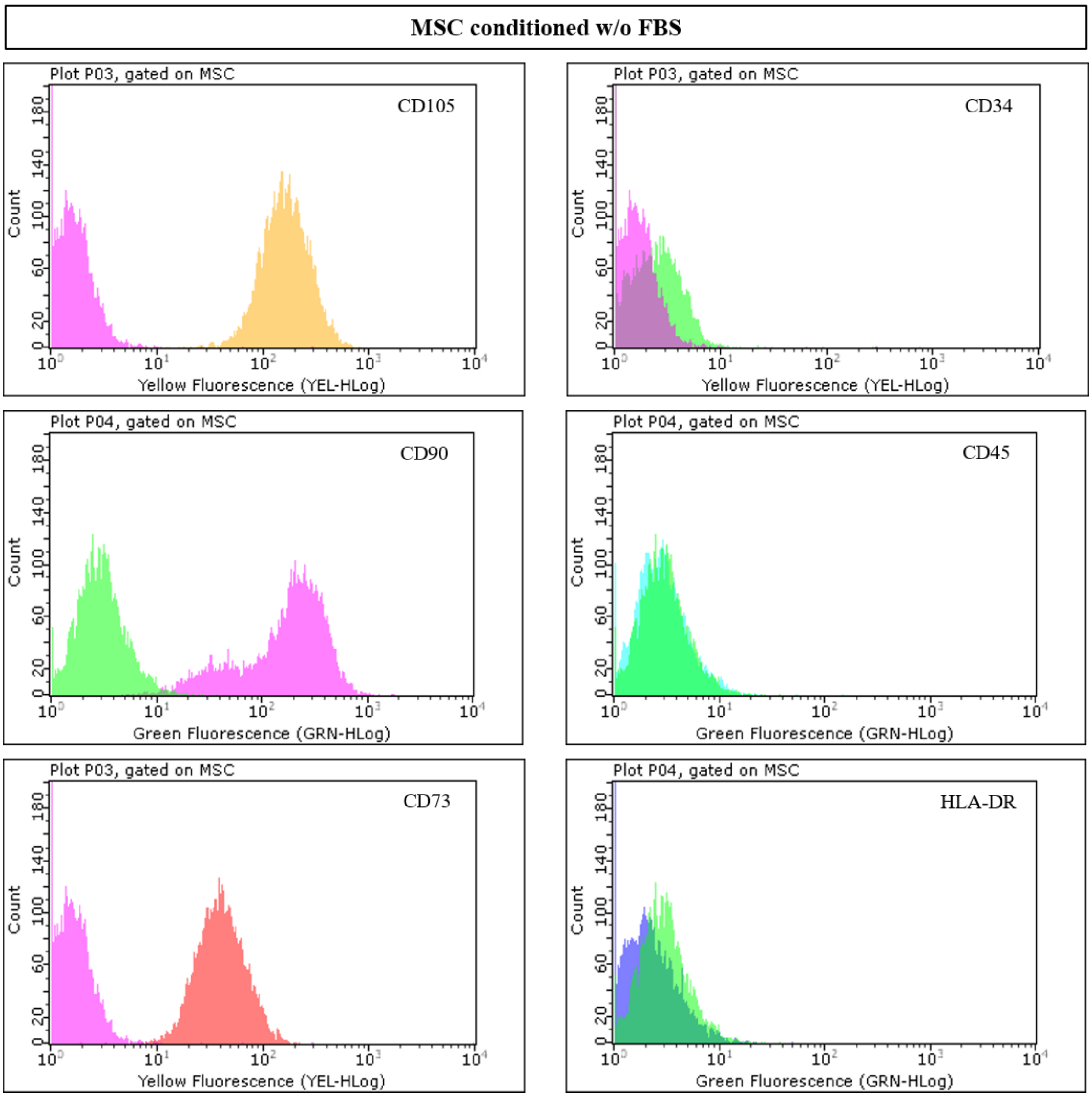

Figure S3: MSC profile by cytometry after conditioning for 48 hours in medium without FBS. A typical profile of MSCs is shown after labelling cells with conjugated monoclonal antibodies: CD105-PE, CD90-FITC, CD73-PE, CD34-PE, CD45-FITC and HLA-DR-FITC.

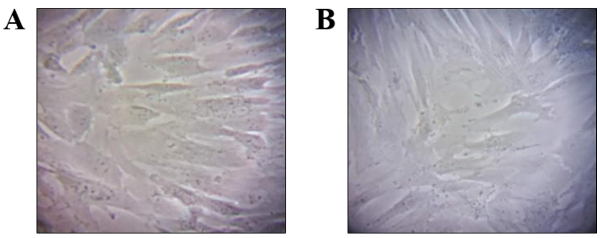

Figure S4: Representative bright-field images of MSCs in culture under all conditions. Bright-field image of MSC cultured in medium with 20% EV-depleted FBS (A) or medium without FBS (B) for 48 h at 200x.

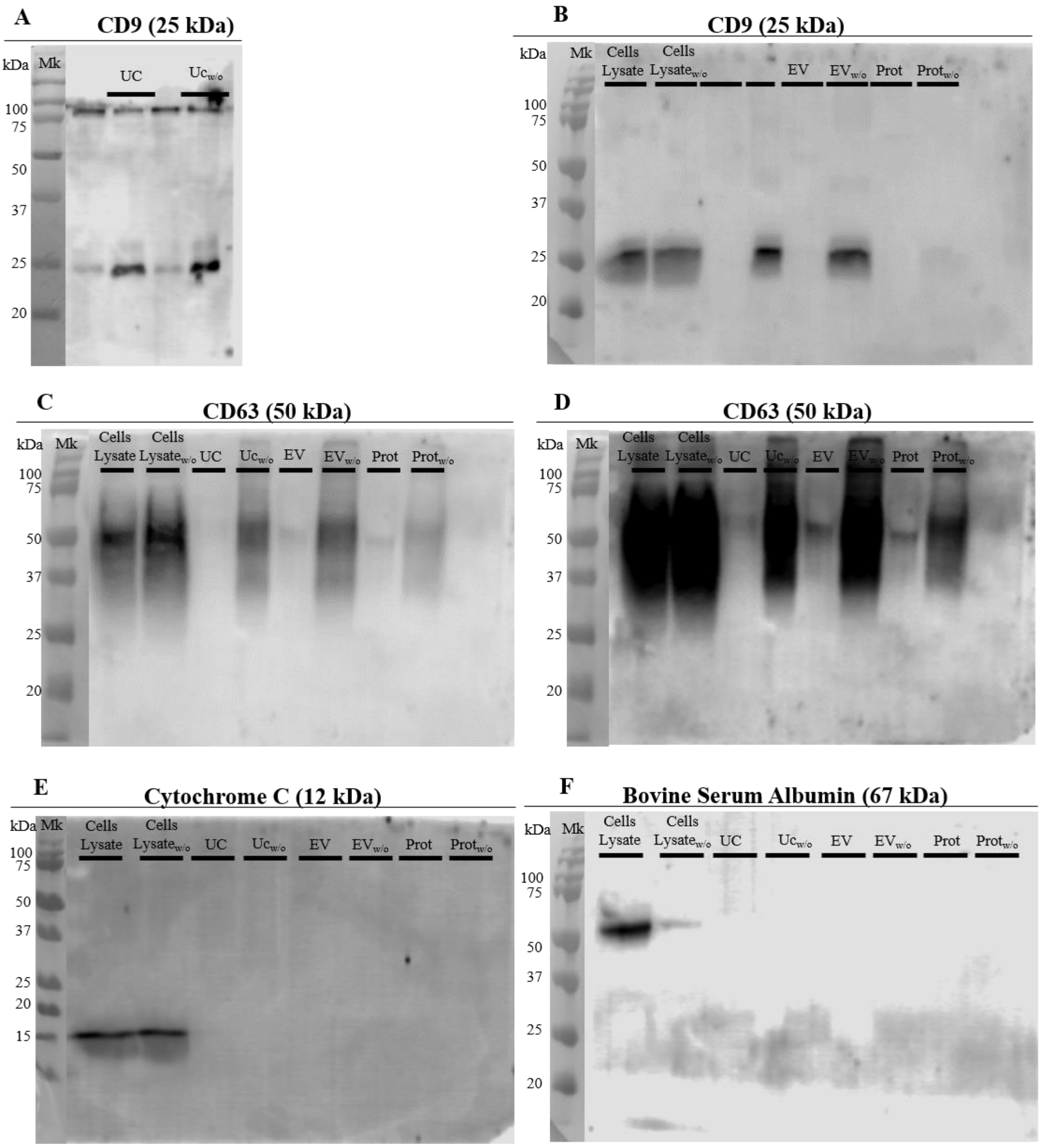

Figure S5: EV markers on UC, $UC_{w/o}$, EV, $EV_{w/o}$, Prot and $Prot_{w/o}$ samples and cell lysates. (A) Presence of CD9 on UC and $UC_{w/o}$ samples. (B) Presence of CD9 in EV, $EV_{w/o}$, Prot and $Prot_{w/o}$. (C, D) Presence of CD63 in all samples at two different exposition times. (E) Absecence of cytochrome C (a mitochondria marker) in all samples, except for cell lysate and cell $lysate_{w/o}$. (F) Absence of bovine serum albumin (as marker of FBS contaminant) in all samples, except for cell lysate from cells growth in medium with FBS.

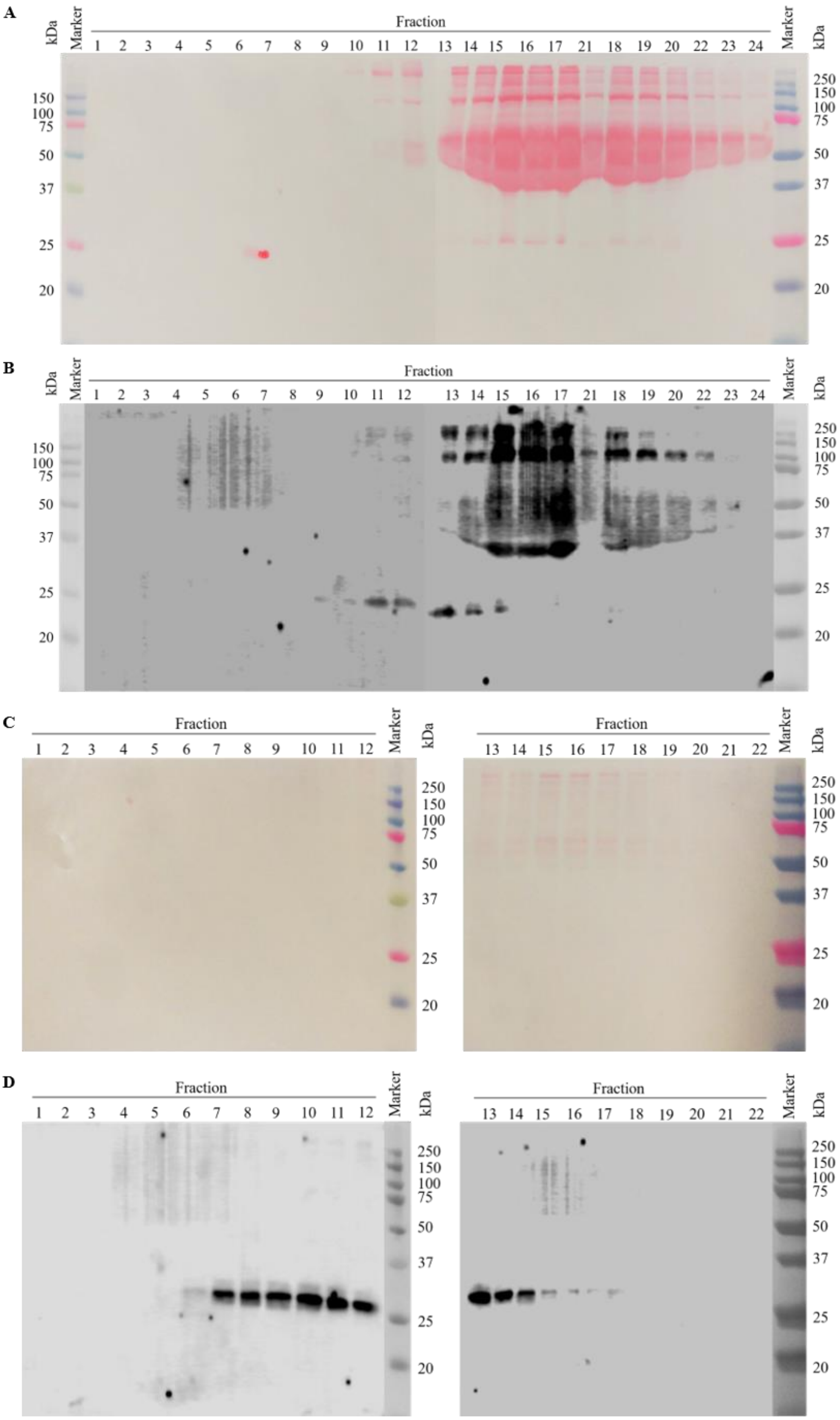

Figure S6: Presence of proteins and EV marker in SEC fractions collected from UC and UC$_{w/o}$ samples. Presence of proteins in nitrocellulose membranes after staining with Ponceau S in fractions eluted from UC samples (A)

and from UC$_{w/o}$ samples (C). Presence of tetraspanin CD9 (24 kDa) in diferent fractions eluted from UC samples (B) and UC$_{w/o}$ samples (D).

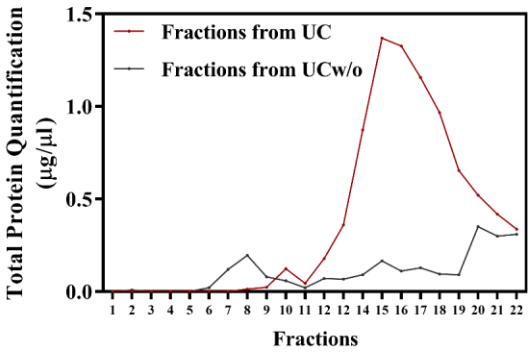

Figure S7: Total protein quantification of SEC fractions collected from UC and UC$_{w/o}$ samples. Protein quantification measuring absorbances at λ = 280 nm of the SEC fractions collected from UC and UC$_{w/o}$ samples.

© 2020 by the authors. Submitted for possible open access publication under the terms and conditions of the Creative Commons Attribution (CC BY) license (http://creativecommons.org/licenses/by/4.0/).
